# Effects of Netarsudil on Actin-Driven Cellular Functions in Normal and Glaucomatous Trabecular Meshwork Cells: A Live Imaging Study

**DOI:** 10.3390/jcm9113524

**Published:** 2020-10-31

**Authors:** Kate E. Keller, Casey Kopczynski

**Affiliations:** 1 Casey Eye Institute, Oregon Health & Science University, Portland, OR 97239, USA; 2 Department of Chemical Physiology and Biochemistry, Oregon Health & Science University, Portland, OR 97239, USA; 3 Aerie Pharmaceuticals, Durham, NC 27703, USA; CKopczynski@aeriepharma.com

**Keywords:** trabecular meshwork, glaucoma, actin cytoskeleton, tunneling nanotubes, extracellular vesicles, live cell imaging

## Abstract

The actin cytoskeleton of trabecular meshwork (TM) cells is a therapeutic target for lowering intraocular pressure (IOP) in glaucoma patients. Netarsudil (the active ingredient in Rhopressa^TM^) is a Rho-associated protein kinase inhibitor that induces disassembly of actin stress fibers. Here, we used live cell imaging of SiR-actin-labeled normal (NTM) and glaucomatous TM (GTM) cells to investigate actin dynamics during actin-driven biological processes with and without netarsudil treatment. Actin stress fibers were thicker in GTM than NTM cells and took longer (>120 min) to disassemble following addition of 1 µM netarsudil. Actin-rich extracellular vesicles (EVs) were derived by two mechanisms: exocytosis of intracellular-derived vesicles, and cleavage of filopodial tips, which detached the filopodia from the substratum, allowing them to retract to the cell body. While some phagocytosis was noted in untreated TM cells, netarsudil potently stimulated phagocytic uptake of EVs. Netarsudil treatment induced lateral fusion of tunneling nanotubes (TNTs) that connected adjacent TM cells; TNTs are important for TM cellular communication. Together, our results suggest that netarsudil may clear outflow channels in TM tissue by inducing phagocytosis and/or by modulating TM communication via EVs and TNTs. These cellular functions likely work together to regulate IOP in normal and glaucomatous TM.

## 1. Introduction

Trabecular meshwork (TM) cells in the anterior of the eye play a key role in the regulation of intraocular pressure (IOP) [1,2]. The actin cytoskeleton provides mechanical support to cells and regulates cellular protrusions, adhesions, and contraction [3]. It is highly dynamic, with the constant addition and removal of actin subunits to filamentous actin (F-actin), which is regulated by a myriad of actin-binding proteins [4]. For instance, phosphorylation of myosin light-chain phosphatase by Rho-associated kinase (ROCK) mediates actin stress fiber assembly and cellular contractility [5]. Inhibition of this Rho-ROCK signaling pathway is an effective method to increase aqueous humor outflow and lower IOP [6,7,8,9,10,11]. Netarsudil is a ROCK inhibitor that has been shown to lower IOP by increasing outflow facility in mouse [12], monkey [13,14], and human [11,15] eyes. In living mouse eyes, optical coherence tomography showed that the TM was widened following application of netarsudil, and there was an increase in the cross-sectional area of Schlemm’s canal [12]. In TM cell cultures, netarsudil effectively reduced actin stress fibers and disrupted focal adhesions, molecular functions compatible with its ROCK inhibitory function [13]. Netarsudil, the active ingredient in Rhopressa™, has received recent FDA approval for the clinical treatment of patients with glaucoma or ocular hypertension [13].

Actin assembles into several structures in TM cells. Linearly arranged actin stress fibers are abundant in cultured TM cells derived from TM tissue of normal and glaucomatous individuals [16,17,18,19,20]. Additionally, fine actin networks are produced, which are characteristic of sheet-like protrusions called lamellipodia [21]. Actin filaments also assemble into cortical actin, networks that lie just beneath the plasma membrane [22]. Actin polymerization in cortical actin networks produces enough force to overcome plasma membrane tension, which is important for many cell processes, including: (1) cell rounding during mitosis; (2) invaginations of the membrane during phagocytic cup formation in phagocytosis; (3) recruiting and translocating cellular vesicles across the plasma membrane during exocytosis; and (4) pushing the leading edge during cell migration [23,24,25,26]. 

Live cell microscopy is a powerful technique to interrogate actin cytoskeleton dynamics and several actin stains are available to researchers [27]. Phalloidin is not useful for live cell imaging due do its low permeability of cell membranes. Lifeact and green fluorescent protein (GFP)-actin are compatible with live cell imaging. However, Lifeact can cause high background fluorescence due to the binding of G-actin and interfere with stress fiber dynamics [28], while GFP may hinder assembly of GFP-tagged monomers into actin filaments [27]. Moreover, GFP-actin requires transfection into cells and, even using a virus-based system, a recent study only achieved a 22% transduction efficiency of TM cells [29]. Due to these limitations, our laboratory has routinely used fluorogenic SiR-actin to label the actin cytoskeleton of TM cells [19,20]. SiR-actin binds to F-actin with high affinity, is readily cell permeable, and is effective at staining cultured TM cells [30]. Using SiR-actin, we investigated highly dynamic filopodia, which emanate from the TM cell surface and contain tight bundles of long parallel F-actin filaments [26]. In certain instances, filopodia that extend between two cells transition to form tubules called tunneling nanotubes (TNTs) [18,31,32,33]. TNTs function in cellular communication and they transport cargoes, such as mitochondria, lysosomes, endosomes, and miRNA. These cargoes are directly transferred between cells without leaving the intracellular environment [31,34]. 

In this study, we investigated the effects of netarsudil on actin-driven biological processes in live normal and glaucomatous TM cell cultures. Our results show that inhibition of the Rho-ROCK signaling pathway disassembles actin stress fibers, as expected, and does not impair TM cell mitosis. Live cell imaging showed that two mechanisms are used to produce actin-rich extracellular vesicles (EVs): exocytosis and cleavage of filopodial tips. While some phagocytosis of EVs was noted in untreated cells, netarsudil potently stimulated phagocytic uptake of EVs. Additionally, we observed that parallel TNTs, drawn out when cells retracted from one another, can fuse laterally with netarsudil treatment. 

## 2. Experimental Section

### 2.1. TM Cell Culture and Actin Staining

Normal (NTM) and glaucomatous TM (GTM) human cadaver eyes were procured from LionsVision Gift, Portland, OR following the rules of the Declaration of Helsinki of 1975. Primary TM cells were cultured from dissected TM following established guidelines [35]. Table 1 shows the demographics of the human donors. These cell strains have been previously characterized by dexamethasone induction of myocilin [19,36]. At least three biological replicates were investigated per experiment. NTM or GTM were plated at 2 × 10^5^ cells per well of a 6-well plate with a 20-mm micro-well #1.5 cover glass as the culture surface (CellVis LLC, Mountain View, CA, USA). Cells were incubated for 16 h and then 100 nM SiR-actin and 10 µM verapamil (Spirochrome, Cytoskeleton Inc, Denver, CO) were added to each well. After 1 h, the media was removed, cells were washed with phosphate-buffered saline, and fresh media (DMEM + 10% FBS) was added to the wells. To test the effects of netarsudil, 1 µM netarsudil (Aerie Pharmaceuticals, Inc., Durham, NC, USA) was added to the media [37]. In some experiments, we compared netarsudil to 1 µM Y27632 (MilliporeSigma, St Louis, MO, USA), another Rho kinase inhibitor [38]. Dimethyl sulfoxide (DMSO) was used as a vehicle control. 

### 2.2. Live-Cell Imaging

To investigate the effects of netarsudil on live TM cell cultures, NTM and GTM cells were imaged at the Advanced Light Microscopy core facility (Oregon Health & Science University, Portland, OR, USA). Images were acquired on a ZEISS Celldiscoverer 7 automated microscope using 625 nm light emitting diode (LED) light to excite the SiR-actin and detecting emission through a 662–756 nm bandpass filter. TM cells were imaged using the 20x objective in combination with the 2x optovar lens, which delivers a nominal 0.95 numerical aperture, and images were captured with a 0.162 micrometer/pixel resolution on a Hamamatsu ORCA-Flash 4.0 V3 camera. Simultaneously, adaptive phase gradient contrast images of TM cells using a far-red LED were acquired. This technique gives superior contrast compared to standard phase contrast. TM cells were imaged every 2 min for the total time stated in the figure legend. This time interval was chosen in pilot studies because 24 fields could be imaged at a time and there was minimal loss of specific SiR-actin labeling due to bleaching, After 60–90 min of no treatment, 1 µM netarsudil or 1 µM Y27632 were added to an injection port and imaging was resumed. The total imaging time was up to 5 h. In all cases, time-lapse movies were made at 3 frames per second using ZEN software (Carl Zeiss Microscopy, LLC, White Plains, NY, USA). 

### 2.3. Confocal Microscopy of Fixed TM Cells

To investigate protein components of EVs, TM cells were grown on collagen type I-coated BioFlex plates (Flexcell International, Inc., Burlington, NC, USA) as described previously [39]. Cells were fixed with 4% paraformaldehyde, blocked with CAS blocking buffer (ThermoFisher Scientific, Waltham, MA, USA), and immunostained with the following primary antibodies: rat monoclonal anti-CD44, clone IM7 (StemCell Tech, Vancouver, BC, Canada), mouse monoclonal integrin alpha v, beta 5, clone P1F6 (Abcam, Cambridge, MA, USA), and rabbit polyclonal anti-ADAMTS4 (Abcam). Secondary antibodies were Alexa Fluor 488-conjugated anti-rat, Alexa Fluor 594-conjugated anti-mouse, and Alexa Fluor 594-conjugated anti-rabbit (ThermoFisher). Coverslips were mounted with ProLong gold anti-fade mounting medium (ThermoFisher) and cells were imaged using a Fluoview FV1000 confocal microscope (Olympus, Waltham, MA, USA). Open-source FIJI software was used to process images post-acquisition [40].

### 2.4. EV Counts and Statistical Analyses

To quantitate phagocytosis, the number of EVs deposited extracellularly were counted in still images of the movies just prior to netarsudil treatment, and at 1 and 2 h post-treatment. Data from NTM (n = 8) and GTM (n = 5) were averaged and a standard error of the mean was calculated. Significance (*p* < 0.05) was determined by a paired *t*-test. 

## 3. Results

### 3.1. Normal and Glaucomatous TM Cells Treated with Netarsudil

**Figure 1:**
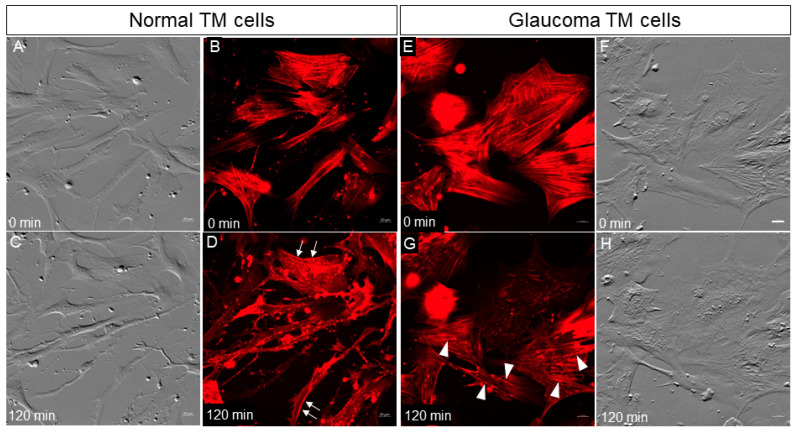
Normal (**A**–**D**) and glaucomatous (**E**–**H**) TM cells before (0 min) and 120 min post-treatment with 1 µM netarsudil. Arrows in panel D show that cortical actin was not affected by netarsudil in normal TM (NTM) cells at 120 min. Arrowheads in panel G show partially disassembled stress fibers in glaucomatous TM (GTM) cells. Red = SiR-actin; grey = phase gradient contrast. Scale bar (Panel F) = 20 µm.

Our previous study showed that GTM cells exhibited thicker actin stress fibers than NTM cells [19]. Thick stress fibers may be more resistant to disassembly by Rho kinase inhibition. Thus, we compared actin stress fiber disassembly in live NTM and GTM cells following treatment with 1 µM netarsudil. At time point 0, NTM cells showed typical appearance by phase gradient contrast (PGC) and abundant linearly arranged SiR-actin-stained stress fibers were visible (Figure 1). Punctate actin-rich vesicles were also obvious both intra- and extracellularly. At 120 min post-treatment, NTM cells showed cell rounding and an increase in long thin filopodial processes emanating from the cell surface (Figure 1C). As expected, netarsudil caused disassembly of the stress fibers, and diffuse intense red patches of intracellular staining appeared (Figure 1D). There was also an increase in the number of punctate actin-rich vesicles. Nearly all of the stress fibers were disassembled by 120 min in NTM cells (Figure 1D). Cortical actin, underlying the cell membrane, was not significantly affected by netarsudil treatment (Figure 1D, arrows). 

In GTM cells at time point 0, actin stress fibers predominated (Figure 1E). Most had a parallel alignment, although some were arranged tangentially. In PGC images, GTM cells did not have the same smooth appearance as NTM cells and striations were observed (Figure 1F). The striations corresponded to where stress fibers were located (Figure 1E,F). Netarsudil treatment of GTM cells induced disassembly of actin stress fibers as expected, but after 120 min, this was not complete. The thickest stress fibers present at time point 0 still remained (Figure 1G, arrowheads). In PGC images, GTM cells did not show as much rounding or phenotypic shape change as NTM cells, but the striations were somewhat diminished (Figure 1H). Thus, disassembly of stress fibers in GTM cells in response to netarsudil treatment was delayed compared to NTM cells. 

**Figure 2:**
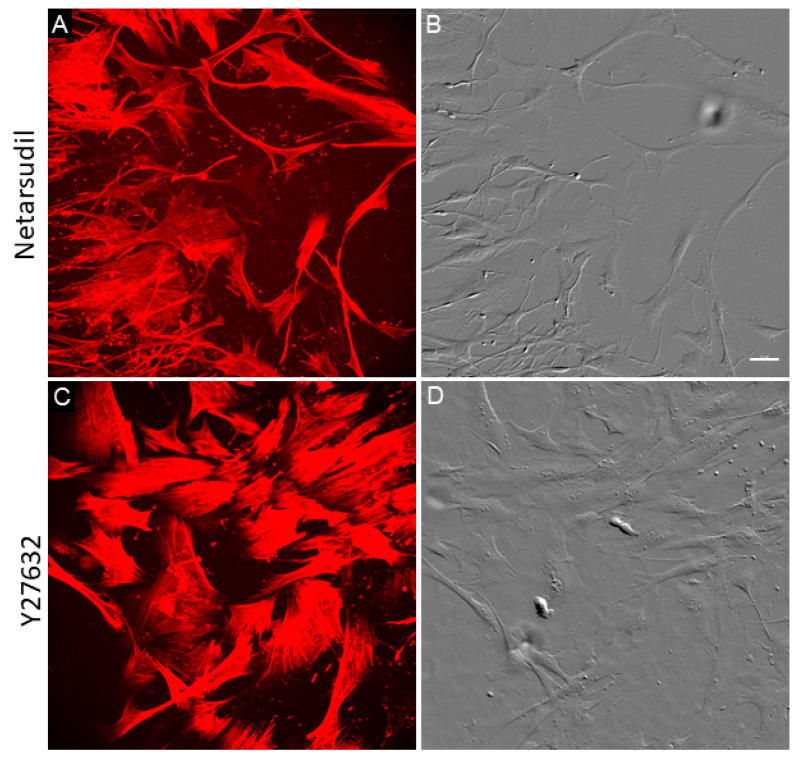
Prolonged effects of netarsudil on glaucomatous TM cells. GTM cells were treated for 3 h with 1 µM netarsudil (**A**,**B**), or 1 µM Y27632 (**C**,**D**), washed and incubated for a further 48 h in fresh medium. Fixed cells were imaged by a laser confocal microscope. Red = SiR-actin; grey = phase gradient contrast. Scale bar = 50 µm.

We also investigated the recovery of TM cells after netarsudil treatment compared to another Rho kinase inhibitor, Y27632 (Figure 2). TM cells were treated with 1 µM netarsudil or 1 µM Y27632 for 3 h, washed, placed in fresh medium without treatment, and allowed to recover for 48 h. As shown in Figure 2, GTM cells treated with 1 µM netarsudil continued to display phenotypic alterations typical of Rho kinase inhibition i.e., long cell processes. Actin stress fibers were present, but these were not as thick as in untreated GTM cells (see Figure 1E). However, GTM cells treated with Y27632 contained abundant thick actin stress fibers following the 48-h recovery period, typical of untreated GTM cells. Thus, the effects of 1 µM netarsudil appeared to be longer lasting than Y27632. 

### 3.2. TM Cell Mitosis

**Figure 3:**
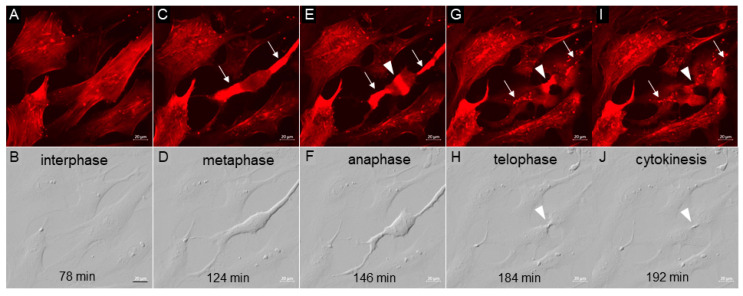
Still images taken from the movie shown in Supplemental Figure S1. The images are from SiR-actin-stained normal TM cells (**A**,**C**,**E**,**G**,**I**) or phase gradient contrast (**B**,**D**,**F**,**H**,**J**). Arrows in (**C**,**E**,**G**,**I**) point to areas of actin staining that appear to be anchor points. Arrowhead in (**E**,**G**,**I**) point to the actin contractile ring, which can also be seen in the phase images (**H**,**J**), where the membrane is invaginated and pinched off to produce two daughter cells. The stages of mitosis (interphase, metaphase, anaphase, telophase, and cytokinesis) and time in minutes are indicated. Scale bar = 20 µm.

Mitosis is a fundamental actin-driven process by which mammalian cells divide. It has been reported that inhibition of the Rho-ROCK signaling pathway using the Rho kinase inhibitor, Y-27632, may affect centrosome separation during mitosis [41]. Here, we observed the actin dynamics in a dividing NTM cell prior to and after the addition of netarsudil (Figure 3; Supplemental Figure S1). NTM cells were imaged every 2 min for 90 min, then 1 µM netarsudil was added and imaging was continued for a further 210 min. The movie shows an NTM cell that initiates mitosis at approximately 75 min (approximately 15 min prior to netarsudil addition) and completes cytokinesis at approximately 200 min (approximately 110 min after netarsudil addition; Supplemental Figure S1). This time frame (~2 h) is typical for mitosis. Still images from the movie are shown in Figure 3. Actin has clear distribution patterns at each stage of mitosis. In interphase, actin stress fibers, cortical actin, and actin-rich punctate vesicles are visible (Figure 3A). In metaphase, which begins at approximately 120 min (approximately 30 min after netarsudil addition), the TM cell starts to round up and F-actin stress fibers disassemble, which is visualized as pools of bright red diffuse actin staining at opposite ends of the cell (arrows; Figure 3C). Cortical actin also depolymerizes, and actin-stained vesicles are not detected. At 140–146 min, a wave of diffuse actin rapidly moves from the pools located at either end of the cell, around the circumference of the cell, and then accumulates in a contractile ring at the center of the cell. The TM cell is now in anaphase (Figure 3E). During the next 20 min, the pools of diffuse actin at each end of the cell start to disseminate and actin-rich vesicles reappear. Concomitantly, two separate cells are now apparent, but these remain connected by a contractile ring of actin. At approximately 184 min (approximately 94 min after netarsudil addition), the TM cell enters telophase, where a furrow in the actin band becomes visible (Figure 3G). This signals the final event of mitosis, cytokinesis, where the two daughter cells separate (Figure 3J). Actin-rich vesicles are again visible as well as some diffuse actin staining. PGC still images are also shown (Figure 3B,D,F,H,J). Together, these data indicate that netarsudil treatment does not significantly affect TM cell mitosis under the conditions studied. 

### 3.3. Extracellular Vesicles (EVs)

**Figure 4:**
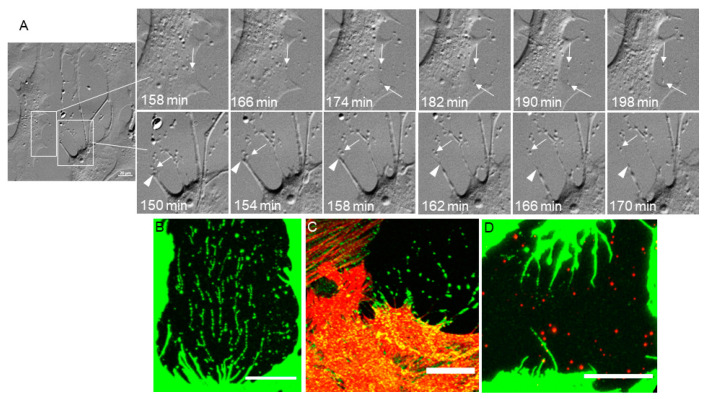
(**A**) Still images taken from the movie shown in Supplemental Figure S2A. Phase gradient contrast images shows two mechanisms of extracellular vesicle (EV) deposition. The top panels show secretion of actin-rich intracellular vesicles from the cell over the course of 40 min (158–198 min). Bottom phase gradient contrast images show the cleavage of filopodial tips as they retract back to the cell over the course of 20 min (150–170 min). Scale = 20 µm. Fixed NTM cells immunostained with (**B**) CD44, (**C**) integrin αVβ5 (green) and SiR-actin (red), and (**D**) ADAMTS4 (red) and CD44 (green). Scale = 20 µm.

Extracellular vesicle (EV) is a catch-all term for any vesicle that is secreted from the cell. EVs include microvesicles (150–1000 nm in diameter) and exosomes (30–150 nm in diameter) [42,43,44]. EVs originate from budding of cell membranes, or by exocytosis, and they contain molecular components (e.g., proteins, DNA and/or RNA) that are tissue, cell, and/or disease specific [42]. EVs have been isolated from the media of TM cell cultures [45,46,47], but the mechanism of origin (exocytosis or membrane budding) has yet to be determined. In our TM cell cultures, actin-rich punctate vesicles are visible on the surface of the coverslip (see Figure 1). Using live cell imaging, we observed the formation of EVs in NTM cells after the addition of netarsudil (Figure 4; Supplemental Figure S2A,B). Two cellular mechanisms that produced EVs were observed: (1) secretion from the cell (exocytosis), which starts at approximately 120 min (bottom left quadrant); and (2) cleavage of filopodia tips (membrane budding), which can be viewed from approximately 150 min (center). Still, PGC images show these mechanisms in more detail (Figure 4A). The top panels show exocytosis as imaged with PGC. Intracellular vesicles move toward the cell membrane. There is a pause and then they are secreted from the cell. The actual secretion is very rapid and is not captured in the 2-min time intervals. Arrows point to the secretion of two vesicles over the course of 40 min. The bottom panels show cleavage of filopodial tips. The filopodial tip is anchored to the substratum, likely via focal adhesions, and the tip is cleaved to leave a small vesicle. This cleavage allows the filopodia to detach from the substratum and retract back to the cell body. The arrowhead points to filopodial tips and the arrow points to the cleaved vesicle. Immunohistochemistry of fixed TM cells shows some molecular components of these EVs (Figure 4B–D). CD44 and integrin αVβ5, a fibronectin receptor [48], leave a ‘trail’ of vesicles (Figure 4B,C), which suggests that a retraction filopodium may have multiple attachment/cleavage events as it returns to the cell body. ADAMTS4, a matrix metalloproteinase (MMP) [39], is also a component of EVs. However, ADAMTS4-stained vesicles are randomly arranged and are not organized in retraction ‘trails’ (Figure 4D). 

### 3.4. Phagocytosis

**Figure 5:**
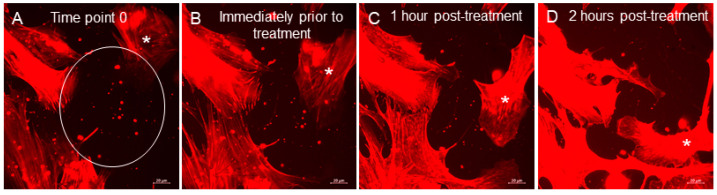
(**A**) Still images taken from the movie shown in Supplemental Figure S3C taken at 1-h time intervals. (**A**) time point 0 with a circle showing the EVs, (**B**) 60 min later, prior to 1 µM netarsudil treatment, (**C**) 1 h post-treatment and (**D**) 2 h post-treatment. An asterisk (*) is used to track movement of one of the cells. Scale bar = 20 µm.

TM cells actively phagocytose debris in aqueous humor [49,50]. Glaucomatous TM cells have decreased phagocytic capacity compared to normal TM cells [19,51]. While many studies have described phagocytosis assays, actin dynamics associated with phagocytosis have not been visualized in live TM cells. NTM cells were imaged for 75 min before 1 µM netarsudil was added and then imaged for an additional 165 min after treatment. At the start of imaging, several groups of actin-rich vesicles were observed extracellularly (Supplemental Figure S3A,B). Before netarsudil treatment, two or three of these vesicles were phagocytosed by the TM cell (Supplemental Figure S3A,B, top right quadrant). However, after netarsudil was added (at 75 min), there was almost immediate activation of phagocytosis in NTM cells. Areas of the cell where fine actin networks predominated were induced to form migratory lamellipodia. These flat sheet-like structures at the leading edge of the cell moved over the surface and started to actively engulf the remaining EVs. This began within 7 min after addition of netarsudil (top right quadrant) and in other areas of the field, where phagocytosis was not observed prior to treatment (top left quadrant at approximately 150 min). A second example shows a similar finding (Supplemental Figure S3C). Some phagocytosis in NTM cells was observed prior to treatment, but netarsudil potently increased lamellipodia formation at approximately 10 min post-treatment (84 min). The lamellipodia migrated toward the EVs and rapidly engulfed them. Phagocytosis can be also be observed in Supplemental Figure S2A,B (bottom left quadrant). Still images from Supplemental Figure S3C are shown in Figure 5. 

We also evaluated how many movies showed active phagocytosis in NTM and GTM cells. For each group, 36 movies were analyzed. Approximately 50% of NTM movies (n = 18) showed phagocytosis, whereas only 22% of GTM movies (n = 8) showed phagocytosis suggesting decreased phagocytosis in GTM cells. To quantitate EV uptake, we counted the number of EVs in NTM and GTM just prior to netarsudil treatment, and at 1 and 2 h post-treatment. As shown in Table 2, there were fewer EVs in GTM cells than NTM prior to netarsudil treatment. Approximately 50% of EVs were engulfed in NTM cells in the first hour, whereas only 25% of EVs were engulfed in GTM cells. However, during the second hour, GTM cells engulfed 50% of the EVs so that by the end of 2 h, the total percentage of EV uptake was similar between NTM (66%) and GTM cells (68%). These data suggest a delayed phagocytic response to netarsudil treatment in GTM cells compared to NTM cells. 

### 3.5. TNT Lateral Fusion

**Figure 6:**
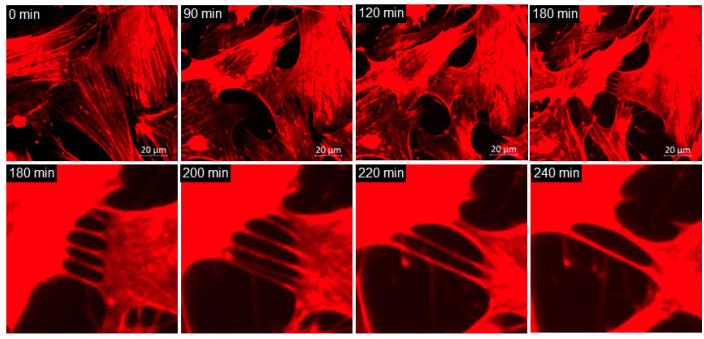
Still images of NTM cells taken from the movie shown in Supplemental Figure S4A,B. Time points shown are 0, 90, 120, 180, 200, 220, and 240 min. Over the course of 60 min, six TNTs undergo lateral fusion so that only two TNTs remain at the final time point. Scale bar = 20 µm.

TNTs are modified filopodia that connect adjacent cells and function in cellular communication. One mechanism by which TNTs form is when short protrusions connecting two cells are drawn out as the cells move away from each other [19,20]. In this study, we describe that TNTs in netarsudil-treated cells can fuse laterally (Figure 6; Supplemental Figure S4). Lateral fusion was not observed in untreated NTM cells. As can be seen in the movie (Supplemental Figure S4A), six TNTs are drawn out between two adjacent NTM cells as they retract from each other (approximately 150 min, top right quadrant). TNT lateral fusion appears to start at the cell surface of one cell and then acts like a zipper along the length of the TNT (Supplemental Figure S4B; center). It is a rapid process with fusion complete within 5 min. Once two TNTs are fused, the resultant TNT is slightly thicker than the original TNTs. A fused TNT can undergo an additional fusion event. Near the end of the movie, two TNTs are fused by being swung into each other. Over 90 min, six original TNT connections were reduced to two as they fused laterally with each other (Figure 6). 

## 4. Discussion

In this study, we investigated the effects of the Rho kinase inhibitor, netarsudil, on actin-driven biological processes in live normal and glaucomatous TM cells. SiR-actin-labeled stress fibers are observed in the majority of untreated primary TM cells in any given microscope field. Previous studies investigated GFP-actin dynamics in transfected porcine TM cells in response to 25 µM Y27632 [29]. The authors showed that Y27632 effectively disassembled actin stress fibers and induced a cell shape change over 30 min post-treatment. Here, we used 1 µM netarsudil to show a similar effect on SiR-actin-labeled stress fibers. These started to disassemble in NTM cells within minutes of treatment and by 2 h, stress fibers were virtually undetectable. Netarsudil also caused a cell shape change, which was previously reported [7,20]. There were subtle differences in actin assemblies in GTM cells. Similar to our prior findings, stress fibers predominated in GTM cells and these were thicker than in NTM cells. Netarsudil induced stress fiber disassembly, but unlike NTM cells, disassembly was incomplete at 2 h [18,19]. Thus, thicker actin stress fibers that are characteristic of GTM cells appear to be more resistant to disassembly by netarsudil. However, it should be noted that stress fibers were lacking in TM tissue in situ, but cortical actin was detected [52]. Unlike stress fibers, cortical actin assembly is governed by mDia1 and the Arp2/3 complex [53], which are not targets of netarsudil. Thus, cortical actin appeared unaffected in netarsudil-treated GTM and NTM cells. The influence of cortical actin on IOP regulation has not been studied in detail. The actin cortex influences membrane shape [22] and is critical for TM cellular functions, such as phagocytosis, endo- and exocytosis. Dynamic remodeling of cortical actin may therefore play an important role in TM-controlled aqueous outflow dynamics. 

Active phagocytosis was associated with regions of fine actin networks in untreated NTM cells. After netarsudil treatment, lamellipodia were induced at these same regions and these sheet-like structures migrated toward EVs, which were then engulfed. Thus, netarsudil increased NTM lamellipodia formation and phagocytosis. It is intriguing that the lamellipodia homed toward EVs rather than other spaces on the coverslip. This suggests EVs may release homing signals or other cues that guide and attract the TM cells. GTM had differences in phagocytosis compared to NTM cells. GTM cells had few cellular regions with fine actin networks and had reduced phagocytosis. Studies on stem cell-derived retinal pigment epithelium (RPE) cells showed that abundant F-actin stress fibers were associated with reduced phagocytic capacity [54]. Our data are consistent with this report as GTM cells had prominent stress fibers with slower turnover than the finer stress fibers found in NTM cells. 

Induction of phagocytosis by netarsudil has not previously been reported. However, other Rho kinase inhibitors (RKI-1447, Y27632, ripasudil, fasudil, PF4950834) have been shown to affect phagocytosis. For instance, RKI-1447 and ripasudil increased phagocytosis in porcine TM cells, as measured by flow cytometry [55,56], and Y27632 restored phagocytosis in stem cell-derived RPE with low phagocytic capacity [54]. Conversely, RhoA activation by lysophosphatidic acid or calpeptin decreased phagocytosis in porcine TM cells [57]. In non-ocular cell types, fasudil promoted phagocytosis in spinal cord-derived microglia and PF4950834 enhanced phagocytosis in alveolar macrophages [58,59]. Thus, it appears that Rho kinase inhibition induces phagocytosis in a wide range of cell types. Disassembly of stress fibers enriches the cellular pool of G-actin available for other cellular processes, such as phagocytosis. In terms of IOP regulation, netarsudil-induced phagocytosis could contribute to IOP lowering by allowing debris to be cleared more effectively from the outflow channels, which could reduce resistance to aqueous flow into Schlemm’s canal. Enhanced phagocytosis of EVs also increases cellular communication between TM cells, and signals related to IOP regulation may be shared. 

Mitosis is highly dependent on the actin cytoskeleton [3]. A contractile ring, composed of actin filaments, forms at the equator of the dividing cell. Previous studies showed that RhoA activity is essential for the formation of the contractile ring [60]. As the ring contracts, it produces a cleavage furrow at the cell equator. Placement of the cleavage furrow depends on the actin anchors in cortical actin [24]. Plasma membrane is pinched and pulled inward during this process, the contractile ring disseminates, and eventually two daughter cells are produced, each bound by its own plasma membrane. These mitotic stages are clearly seen in our PGC- and SiR-actin-labeled TM cells. Despite the critical role of RhoA in contractile ring formation [60], addition of netarsudil does not appear to disrupt mitosis in TM cells. It is worth noting that Rho kinase is only one of several downstream effectors of RhoA activity [61], and it is possible that for the critical process of mitosis, there are other kinases that can substitute for the loss of Rho kinase activity. Indeed, the disassembly of actin stress fibers caused by Rho kinase inhibition may increase the free actin pool and indirectly benefit contractile ring formation. 

EVs are secreted membrane vesicles that include exosomes (50–150 nm diameter) and microvesicles (150–1000 nm in diameter) [42,43]. Many different sizes of EVs are present on the surface of our coverslips, suggesting that both of these types of EVs are secreted by TM cells. EVs are utilized for cellular communication and they contain a variety of proteins, mRNAs, DNA, and microRNAs (miRNAs) [42,43]. Often, EVs contain cell type-specific molecular signatures and these can vary by disease status. For instance, EVs isolated from normal human aqueous humor contain miRNAs [62], while those in glaucomatous aqueous humor contain a different miRNA signature [63]. Our live cell imaging shows that intracellular-derived vesicles are secreted from the cell and other EVs are derived from cleavage of filopodia tips. While the movies showing production of EVs are derived from TM cells treated with netarsudil, EVs are also present in untreated cell cultures so it is not a netarsudil-dependent event. However, it is possible that generation of EVs may be stimulated by netarsudil due to the excess pool of G-actin available for remodeling actin assemblies. The deposited EVs showed positive immunoreactivity for CD44, integrin αVβ5, and ADAMTS4. Because CD44-positive and integrin αVβ5-positive EVs were arranged in ‘trails’, these are likely derived from cleavage of filopodial tips as they retract to the cell body. EVs are known to contain matrix metalloproteinases (MMPs) [64,65], and TM cell exosomes contain MMP-2 [47]. Here, we show that an additional MMP, ADAMTS4, immunolabels EVs from TM cells. While other molecular components of EVs are yet to be elucidated, it seems likely that important signals are being communicated between TM cells via EVs and phagocytosis. Moreover, the molecular signatures in EVs derived from the two sources, exocytosis or cleavage of filopodial tips, may differ and influence the identity of signals communicated. Furthermore, EVs may differ in normal and glaucoma TM cells. For instance, the ECM deposited by GTM cells is different than that of NTM cells [66], and thus cleavage of GTM filopodial tips likely produces EVs with different ECM protein signatures than NTM cells. Since cellular communication is essential to coordinate cellular responses to IOP in vivo, the molecular content of EVs may influence IOP regulation. 

Our live imaging study revealed that TNTs can fuse laterally. To date, two models of TNT formation have been proposed: (1) the tips of two filopodia protruding from opposing cells touch and then fuse to form a tubular connection; and (2) short protrusions between adjacent cells are drawn out as the cells move away from each other [31,32]. Our previous work suggests that both mechanisms of TNT formation are utilized by normal and glaucomatous TM cells [19,20]. Here, we observed six TNTs formed between two NTM cells retracting from one another. These parallel TNTs then fused together laterally. In most cases, the lateral fusion was initiated at the cell surface of one of the cells and then the TNTs ‘zippered’ toward the other cell. This suggests that molecular machinery at the actin cortex may be involved in initiation of lateral fusion. However, one fusion event involved two ‘rope-like’ TNTs being swung into each other. It is currently unclear what molecular mechanisms govern this process. TNTs function in cellular communication [31] and lateral fusion of TNTs may allow the transport of larger cargoes, or an increased number of cargoes, since each of the fused TNTs was slightly thicker than the TNTs from which they were derived. Alternatively, TNT fusion may maintain the connections for an extended period of time. Previously, we showed that TNTs formed by GTM cells had different physical properties than TNTs formed by NTM cells, and that transfer of fluorescently labeled vesicles was delayed in GTM cells [19]. Thus, alterations to TNT formation may enhance or delay propagation of IOP-related signals in situ. 

Infrequently, we observed highly motile cells within a primary TM cell culture. Recent studies using single-cell RNASeq have demonstrated that there are approximately 12–19 distinct cell types in TM tissue [67,68]. The major cell types in the conventional TM outflow pathway are fibroblast-like TM cells (TM1), myofibroblast-like TM cells (TM2), lymphatic-like cells, vascular endothelial cells, and macrophages [67]. Our primary cell cultures were derived from dissected TM tissue and are likely to be predominantly TM1 and TM2 cells from beams/cribriform tissue, with smaller proportions of juxtacanalicular TM cells, lymphatic-like cells, and macrophages. Our prior studies show our NTM and GTM cells induce myocilin protein in response to dexamethasone treatment consistent with TM1 and TM2 cells [19,36]. It is unlikely that Schlemm’s canal endothelial cells are present as these slow-growing cells are usually displaced by faster growing TM cells [69].

There are several limitations of this study. Since SiR-actin is a jasplakinolide analog, there remains a possibility that SiR-actin may stabilize actin filaments. A direct comparison of 100 nM SiR-actin with 100 nM jasplakinolide showed no detrimental effects on actin stabilization [30]. Furthermore, 100 nM jasplakinolide had no effect on outflow facility in monkey eyes [70]. Our prior studies using Y27632 [20], and the netarsudil results shown here, indicate that SiR-actin-labeled stress fibers readily disassemble. Moreover, SiR-actin labeling does not interfere with normal TM biological processes, such as mitosis or phagocytosis, which are dependent on highly dynamic actin filaments. Thus, we conclude that SiR-actin is a useful fluorescent stain for monitoring actin dynamics in live TM cells. Our results indicate that netarsudil effects were prolonged compared to a similar concentration of Y27632. This could result from the greater potency of netarsudil compared to Y27632 (2 nM vs. 300 nM Ki for ROCK2, respectively [37,38]), differences in their physicochemical properties (e.g., XLOGP3 4.6 vs. 0.9, respectively [71,72]), or both. Another limitation of this study is that we investigated TM cells cultured on glass coverslips, which is a non-native environment. Glass has a Young’s modulus of around 70 GPa, which is a million times stiffer than that of normal TM tissue (~4 kPa) [73]. Cells cultured on stiff substrates assemble stress fibers, as is observed here. Thus, stress fibers may be an artefact of the cell culture environment. In this regard, a recent two-photon microscopy study failed to detect actin stress fibers in human TM tissue in situ [52]. Future studies will analyze TM cells cultured on substrates mimicking the stiffness of NTM (4 kPa) and GTM (75 kPa) tissue [73].

## 5. Conclusions

In conclusion, live cell imaging is a powerful technique to decipher TM cellular behaviors that may not be apparent in fixed cells. Several novel findings were revealed by this study: cellular regions with fine actin networks are associated with phagocytosis, the Rho kinase inhibitor netarsudil enhances phagocytosis, EVs are produced by exocytosis as well as by cleavage of filopodial tips, and TNTs can fuse laterally. Phagocytosis and cellular communication via EVs and TNTs likely work in conjunction to facilitate IOP homeostasis in normal and glaucomatous TM. 

## Figures and Tables

**Table 1:** Demographics of human donor eyes.

Cell Strain	Sex	Age	Cause of Death	Glaucoma?
NTM 2018-0070	M	54	Cardiac arrest	No
NTM 2018-1341	M	55	Myocardial infarction	No
NTM 2018-1233	M	53	Lung cancer	No
NTM 2012-1457	M	47	Ventricular fibrillation arrest	No
NTM 2007-0125	M	4	Respiratory failure	No
NTM 2011-1808	M	19	Multiple trauma	No
GTM 2018-1672	M	57	Respiratory failure	Yes
GTM 2018-0374	M	79	Ischemic cerebrovascular accident	Yes
GTM 2017-1729	F	64	Anoxic brain injury, cardiopulmonary arrest	Yes

**Table 2:** Number of EVs in NTM and GTM cells immediately prior to treatment, and at 1 and 2 h post-treatment.

	NTM	GTM
# of movies analyzed	8	5
# of EVs prior to Netarsudil treatment	18.25 ± 4.4	9.4 ± 3.1
# of EVs 1 h after Netarsudil treatment	9.25 ± 2.8	7 ± 2.7
# of EVs 2 h after Netarsudil treatment	6.13 ± 2.1	3 ± 0.9
% of EVs engulfed (prior vs. 1 h)	49.3%	25.5%
% of EVs engulfed (1 h vs. 2 h)	33.7%	57.1%
Total EVs engulfed (prior vs. 2 h)	66% *	68% **

Data show mean # of EVs ± standard error; * *p* = 0.021; ** *p* = 0.004 by paired *t*-test.

## Supplementary Materials

The following are available online at https://www.mdpi.com/2077-0383/9/11/3524/s1. Video S1: Live cell movie showing TM cell mitosis in SiR actin-stained cells. NTM cells were imaged every 2 min for a total of 5 h. The cells were untreated for the first 90 min, and then 1 µM netarsudil was added for the remaining 210 min. Mitosis starts at approximately 75 min. Scale bar = 20 µm. Video S2: Live cell movie showing two mechanisms of extracellular vesicle (EV) deposition by NTM cells. (S2A) Phase gradient contrast movie and (S2B) SiR-actin staining. EV deposition by exocytosis is seen just to the left of center, starting at approximately 120 min, while cleavage of filopodia tips is observed from approximately 150 min. TM cells were imaged every 2 min for a total of 5 h. The cells were untreated for the first 90 min, and then 1 µM netarsudil was added for the remaining 210 min. Scale bar = 20 µm. Video S3: Normal TM cell phagocytosis. (S3A) phase gradient contrast; (S3B,C) SiR-actin stained NTM cells. TM cells were imaged every 2 min for a total of 4 h. The cells were untreated for the first 75 min, and then 1 µM netarsudil was added for the remaining 2 h 45 min. Scale bar = 20 µm. Video S4: Live cell movie showing TNT lateral fusion in SiR-actin labeled TM cells. (S4A) TM cells were imaged every 2 min for a total of 4 h. The cells were untreated for the first 75 min, and then 1 µM netarsudil was added for the remaining 2 h 45 min. TNTs are drawn out between two cells around 150 min at the top left and lateral fusion can be seen at approximately 180 min. (S4B) A cropped area of the movie shown in 4A, which starts from 75 min, immediately after netarsudil treatment. Scale bar = 20 µm.
